# The role of natural killer cell in gastrointestinal cancer: killer or helper

**DOI:** 10.1038/s41388-020-01561-z

**Published:** 2020-12-01

**Authors:** Feixue Wang, Jennie Ka Ching Lau, Jun Yu

**Affiliations:** 1 Institute of Digestive Disease, Department of Medicine and Therapeutics, State Key Laboratory of Digestive Disease, Li Ka Shing Institute of Health Sciences, CUHK Shenzhen Research Institute, The Chinese University of Hong Kong, Hong Kong SAR, PR China; 2 Faculty of Medicine, SHHO College, The Chinese University of Hong Kong, Hong Kong SAR, PR China

**Keywords:** Gastric cancer, Liver cancer

## Abstract

Gastrointestinal cancer is one of the leading health problems worldwide, with a high morbidity and mortality. To date, harnessing both the innate and adaptive immune system against cancer provides a selective and effective therapeutic strategy for patients. As a first line defense against cancer, natural killer (NK) cells can swiftly target and lyse tumor cells without prior activation. In addition to its pivotal role in innate immunity, NK cells also play unique roles in the adaptive immune system as it enhance anti-tumor adaptive immune responses through secretion of cytokines and retaining an immunological memory. All these characteristics make NK cell a promising anti-cancer agent for patients. In spite of scarce infiltration and impaired function of NK cells in tumors, and the fact that tumors easily develop resistant mechanisms to evade the attacks from endogenous NK cells, multiple strategies have been developed to boost anti-tumor effect of NK cells and abolish tumor resistance. Some examples include adoptive transfer of NK cells after ex vivo activation and expansion; restoration of NK cell function using immune checkpoint inhibitors, and monoclonal antibody or cytokine treatment. Preclinical data have shown encouraging results, suggesting that NK cells hold great potential in cancer therapy. In this review, we discuss NK cells’ cytotoxicity and modulation function in GI cancer and the current application in clinical therapy.

## Introduction

Gastrointestinal (GI) cancer, referring to the malignant condition arising in the digestive system, is one of the most serious health problems worldwide. According to global epidemiological data (2018), among all the cancer types, colorectal cancer (CRC) is the fourth (6.1%) most common diagnosed cancers and second leading cause (9.2%) of cancer-related deaths, followed by gastric cancer (5.7%) and liver cancer (4.7%) for incidence, and gastric cancer (8.2%) and liver cancer (8.2%), esophagus cancer (5.3%) for mortality [1]. Surgical resection remains the mainstay of treatment for early-stage cancer. However most patients are already at a late stage disease at initial diagnosis. Current therapeutic strategies for late stage GI cancers include radiotherapy, chemotherapy, and targeted therapy, but are far from satisfactory with poor clinical response and high risk of therapeutic toxicity. Therefore, there is an urgent need to develop novel strategies to improve the therapeutic efficiency and clinical prognosis of patients with GI cancer.

To date, immunotherapy has come into stage with the great success of immune checkpoint inhibitors represented by the anti-programmed cell death protein 1/ programmed death-ligand 1 (anti-PD-1/PD-L1) and anti-cytotoxic T-lymphocyte-associated protein 4 (anti-CTLA-4) in multiple cancers. Low cytotoxicity, long-lasting tumor regression, and recurrence prevention make this novel therapeutic strategy a promising candidate for cancer treatment. However, translation of this success to GI cancer is not as satisfactory as in melanoma and lung cancer. In the Phase 1 and 2 clinical trials of CRC and advanced pancreatic cancer, few patients showed responses [2]. Only a limited number of patients with special characteristics achieved benefits compared with those undergoing traditional chemotherapy. It is assumed that scarce T-cell infiltration, poor effector T-cell responses, and adaptive resistance to ICIs are main reasons for this failure. Nevertheless, it paves way for harnessing the immune system in cancer therapy. In addition to the cytotoxic T lymphocytes, many other immune cell types in both the adaptive and innate immune systems have gained interests in the cancer immunotherapy, among which NK cell is one of the promising candidates [3].

Discovered in the 1970s, natural killer cell (NK cell) received its name for the inherent ability to kill viruses and tumor cells rapidly. NK cells play a predominant role in cancer immunosurveillance [4–6]. Recently, multiple basic research studies along with sing-cell RNA sequencing technologies unveiled the similarities between NK cells and cytotoxic CD8^+^ T cells ranging from phenotype to function [7]. Aside from the natural cytotoxicity towards transformed cells, NK cells can also secrete multiple cytokines and chemokines, which can further modulate the immune microenvironment. All these characters suggest that NK cells hold great potential in cancer therapy. In this review, we summarized NK cells’ functions and current clinical applications in cancer, with a focus on the GI cancers.

## Overview of NK cell

The NK cell first came into knowledge in 1970s when researchers found that a type of large granular lymphocyte isolated from mice spleen can lyse several tumor cell lines rapidly (within one to four hours) [8]. Although NK cells belong to the lymphocytes family due to their morphology, they are also classified as part of the innate immunity, as they recognize targets through an array of germline-encoded receptors instead of antigen receptors generated by V(D)J recombination and they do not express the CD3 subunits as in T lymphocytes.

NK cells, typically identified as CD3^-^CD56^+^ cells in human and CD3^-^NK1.1^+^ in several mouse strains, account for about 5–15% lymphocytes in the circulation [9]. Aside from peripheral blood, NK cells are also found in non-lymphoid tissues such as liver, uterus, adipose tissue and gut [10]. In humans, NK cells can be divided into two subsets based on the CD56 expression level, the CD56^dim^ (~90%) and CD56^bright^ (~10%), mirroring the functions of cytotoxic and helper cells respectively [11].

### NK cell activation—“missing self” and “induced self”

Ten years after the discovery of NK cells, the “missing self” hypothesis postulated by Kärre et al. [12] gave us a much clearer knowledge of NK cell recognition. According to this hypothesis, killer-cell immunoglobulin-like receptors (KIRs) expressed on the NK cell surface can recognize major histocompatibility complex I (MHC I), which is widely expressed on the surface of normal cells and exerts an inhibitory signal to protect the healthy cells from NK cell attack. However, in cells undergoing virus infection or malignant transformation, the MHC-I expression is decreased or even lost, such that NK cells can be relieved from KIR-induced inhibition and exert their lysis function. Aside from the “missing self” mechanism, the “induced self” is also proposed in the NK cell-mediated immune surveillance. The activating receptors on NK cells including the natural cytotoxicity receptors (NCRs) and natural killer group 2D (NKG2D) can recognize the ligands on the stressed cells and deliver the activating signal [13]. These two mechanisms involve the inhibitory and activating receptors respectively, the balance of which determines the NK cell function status.

### NK cell function – killer and helper

Once activated, NK cells exert lysis function in different ways. Firstly, they can release lysis granules with perforin and granzyme in it [14]. Then, the death receptors such as Fas ligand (FasL) and tumor necrosis factor-related apoptosis-inducing ligand (TRAIL) are also contributors to NK cell-mediated cytotoxicity [15]. Additionally, NK cells can recognize and induce the lysis of antibody-coated targets through CD16 (FcγRIIIa), which is highly expressed on the CD56^dim^ subset, a process referred to as antibody-dependent cell cytotoxicity (ADCC) [16]. Aside from the direct cytotoxicity, NK cells can also modulate both innate and adaptive immunity through secreting an array of cytokines, growth factors, and chemokines [17]. For example, NK cells are now regarded as the major producer of interferon-γ (IFN-γ), which plays a critical role in shaping the T-cell response, including the T_H_1 polarization and CD8^+^ T-cell activation [18]. They can also secrete chemokines such as CCL3 and CCL4 that can help recruit other immune cells to the inflammation site to enhance the immune response.

## Multifaced NK cell in GI cancer

**Fig. 1:**
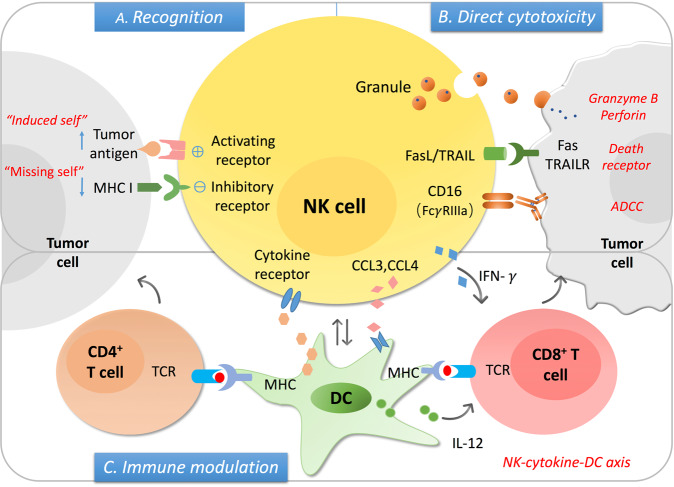
The NK cell function mechanism in cancer. The NK cell can function as the innate cytotoxic effector as well as a regulator modulating both the innate and adaptive immunity. **a** As the innate killer cells, the stress-induced ligands (“induced self”) and decreased MHC I expression (“missing self”) level can be recognized by the activating and inhibitory receptors on NK cells respectively, the balance of which determines the NK cell activation status. **b** After activation, NK cells can induce the lysis of the target cells via secreting granules with granzyme B and perforin, the death receptor/ligand interaction and antibody-dependent cell cytotoxicity. **c** On the other hand, activated NK cells can secrete an array of cytokine and chemokine that promoting the DC maturation and recruitment, which can further modulate T-cell response including the cytotoxic CD8^+^ T-cell and Th cell activation.

The exact role of NK cell in GI cancer was initially deciphered by retrospective analysis of clinical data and utilizations of animal models. In an 11-year follow-up study based on the general population (n = 3625), the infiltration and cytotoxicity of NK cells were found to be closely correlated with cancer risk, implying a functional role of NK cell in tumorigenesis [19]. Then in CRC and HCC, by analyzing NK cells in tumor tissues and peripheral blood, the decreased frequency of NK cell was found to be associated with higher risk of cancer and worse clinical outcome [20]. This is further supported by the findings that depletion of NK cells by performing antibody-mediated methods or using transgenic mice model (Nfil3^-/-^ mice) vastly exacerbated tumorigenesis in mouse models developing malignant diseases [21]. Many mechanisms have been put forward for understanding the NK cell-mediated tumor surveillance. According to the biological function of NK cells, both the direct cytotoxicity and immune modulation effect are involved in NK cell mediated anti-tumor effects in GI cancers (Fig. 1).

### NK cell function as innate effector in GI cancer

As NK cells can respond to the stimulation within hours without pre-immunization, it is commonly regarded as the first-line fighter against cancer. In this process, NK cells act as effector cells. Both the reduced MHC-I and stress-induced specific ligands expressed on the transformed cells can be recognized by surface receptors, leading to the activation of NK cells (Fig. 1). Currently, NK cell activation through the major activating receptor NKG2D is the most well-characterized mechanism in tumor surveillance. A positive correlation of NKG2D expression level and clinical survival is observed in gastric cancer, and in vitro experiments confirmed the NK cell cytotoxicity towards gastric cancer cell line [22]. Moreover, using human pancreatic cancer cell lines and orthotopic pancreatic cancer models, NK cells were found to be cytotoxic to cancer stem cells (CSCs) through the NKG2D-dependent recognition of the CSCs markers such as CD133 and CD24, highlighting the promising role of NK cells in the control of tumor recurrence and metastasis [23]. In addition, the death receptors also contribute to NK cell-mediated anti-tumor cytotoxicity. NK cells can induce significant apoptosis of HCC cell line Hep3B through TRAIL/TRAIL receptor (tumor necrosis factor-related apoptosis-inducing ligand) interaction [24]. Same mechanism was also involved in the NK cell-mediated suppression of liver metastasis in CRC [25]. Similarly, in MC38 (murine primary colon carcinoma cell line)-based liver metastasis of CRC mice model, Nlrp3 inflammasome can increase the IL-18 secretion and promote the maturation of hepatic NK cells with increasing FasL expression, the Fas/FasL interaction can exert cytotoxicity towards tumor cells [26].

Interestingly, some anti-tumor chemotherapeutic agents have been shown to exert tumor-suppressive effect through NK cells. For example, the therapeutic monoclonal antibodies such as Cetuximab (anti-epidermal growth factor receptor/EGFR antibody), Herceptin/Trastuzumab (anti-human epidermal growth factor receptor 2/HER2 antibody), and Rituximab (anti-CD20 antibody) are demonstrated to function in patients partly through NK cell-mediated ADCC [27]. Cetuximab is now regarded as a standard choice for metastatic CRC. By analyzing the immune cells of peripheral blood mononuclear cell (PBMC) derived from CRC patients, NK cells were found to be activated upon Cetuximab treatment [28]. Moreover, a higher basal NK cell cytotoxicity indicates better therapeutic response [29], suggesting the possible involvement of NK cell in the cetuximab mediated tumor suppression.

### NK cell function as a helper in GI cancer

Accumulating evidence suggests that NK cells are more than just innate immune cells [30]. Firstly, NK cells can shape the adaptive immunity through secreting cytokines that act on other immune cells such as dendritic cells (DCs) [31], neutrophils [32], T cells [33]. Secondly, emerging evidence has shown that NK cells can acquire immunological memory, a feature of adaptive immunity [4]. Finally, the similarities between NK cell and CD8^+^ T-cell are identified by basic research as well as single-cell RNA sequencing technologies. Intriguingly, through analyzing the whole-genome microarray data sets of the Immunological Genome Project, a NK-T-cell complex was observed, indicating the close transcriptional relationship between NK cell and T cell [7]. Now there is a consensus that NK cell is a bridge linking innate and adaptive immunity, in which cytokine production is the major contributor. The modulation function also plays a vital role in NK cell mediated tumor surveillance.

Dendritic cells (DC), a type of antigen presenting cell (APC), is an important player in the NK cell-mediated shaping of the adaptive immunity via NK-cytokine-DC axis (Fig. 1). NK cell can promote DC recruitment and maturation; reciprocally, DCs can also help prime NK cells and enhance the cytotoxicity [34]. For example, NK cells can recruit DCs into tumor microenvironment and enhance the anti-tumor immune activity through secreting CCL5 and XCL1 [31]. In addition, NK cells can also promote the antigen cross presentation of DCs, which can induce cytotoxic CD8^+^ T-cell response [35]. Similar mechanism was shown in MHC I^low^ cell line A20 and CT26 that activated NK cell can recruit the DC to induce protective CD8^+^ T-cell response [36]. The immunoadjuvant toll-like receptor 5 (TLR5), Entolimod, which has already been used in clinical practice, can exert anti-metastatic effects and keep immune memory in CT26 tumor cell induced CRC model through the NK-DC-CD8^+^ T-cell axis [37]. Recently, scientists found that the NK-DC axis is associated with anti-PD1 immunotherapy responsiveness. For example, in melanoma it was found that the frequency of NK cells is correlated with the abundance of protective DCs, the ICI treatment response as well as the overall survival, in which the NK cell production of cytokine FLT3LG plays a pivotal role [38].

### Impaired NK cells in GI cancer

Tumor evasion from immune response is regarded as a hallmark of cancer. Extensive studies have found scarce infiltration and impaired function of NK cells in GI cancer. Disrupted balance of inhibitory and active receptors often underlies NK cell dysfunction. The significantly reduced expression of activating receptor NKG2D on NK cells isolated from PBMC and tumor tissue is demonstrated in patients with gastric cancer [39], CRC [40], and HCC [41]. Shedding of the major histocompatibility complex (MHC) class I chain-related protein A and B (MICA/B), the ligands of NKG2D that can be expressed on tumor cells, is one of the most characterized mechanism responsible for the impaired NKG2D recognition and subsequent tumor immune evasion [41, 42]. Moreover, the inhibitory receptors such as PD-1 were significantly increased in tumor tissues derived from digestive cancer patients, including the esophageal squamous-cell carcinomas (ESCC), HCC, CRC, GC, and biliary cancer [43]. In addition to PD-1, many other immune checkpoints have also been identified in NK cells. The expression of T-cell immunoglobulin mucin-3 (Tim3), natural killer group 2 member A (NKG2A) and T-cell immunoglobulin and ITIM domain (TIGIT) on NK cells are increased in the tumor tissue and is correlated with impaired NK cell cytotoxicity, advanced disease stage and poor survival in CRC, GC and HCC patients [44, 45]. Recently, a novel inhibitory receptor CD96 is found to be upregulated in intratumoral NK cells from HCC patients. CD96^+^ NK cells are responsible for impaired IFN-γ production and predict poor clinical outcomes [46]. NKG2A, TIGIT, CD96 along with the PD-1 are now well acknowledged immune checkpoints on NK cells, suggesting that they are potent candidates for cancer immunotherapy.

Conversely, there are certain types of NK cells exerting tumor-promoting effect in GI cancer. In CRC patients, the tumor-associated NK cell (TANK) was found to make contributes to the tumor angiogenesis and invasion through secreting proangiogenic factors and tissue remodeling/invasion factors secretion via STAT3 and STAT5 pathway [47, 48].

The tumor microenvironment (TME) is the major contributor for the impaired NK cell function in cancer. Firstly, tumor cells, stromal cells, and other kinds of immune cells in TME can influence NK cells’ function. Release of MICA from tumor cells can significantly suppress the NKG2D expression on NK cells in HCC patients [41]. Moreover, tumor-associated fibroblast can also inactivate NK cell through the Indoleamine 2,3-dioxygenase (IDO) and prostaglandin E-2 (PGE) secretion [49]. The immune-suppressive cells such as regulatory T cells (Tregs), myeloid-derived suppressor cells (MDSCs), and neutrophils were also found associated with NK cell dysfunction via direct cell contact [50–52]. Secondly, the physical factors that provide cells with basic nutrition and environment are also contributors for NK cell dysfunction. Take oxygen as an example, intratumoral hypoxia is a very common phenomenon in cancer. Hypoxic tumor-derived microvesicles (TD-MVs) can decrease the NKG2D expression of NK cells via miR210 and miR23a [53]. Moreover, the hypoxia-induced autophagy in tumor cells can help the transformed cells escape the NK cell attack by degrading the effector molecular granzyme B [54, 55]. The pH alteration is another important factor. It has been found that tumor-derived lactate can decrease the pH level and induce apoptosis of NK cells, resulting in low infiltration of NK cells in liver metastasis of CRC [56].

Taken together, NK cells play multiple roles in the tumor microenvironment (Fig. 1). As the innate immune cell, it can exert killer function towards the stressed or transformed cells. On the other hand, it can be a helper modulating both the innate and adaptive immunity through cytokine and chemokine secretion. However, under the effect of specific tumor microenvironment, the failure of NK cell infiltration into tumor cells and impaired cytotoxicity are frequently observed in cancer. From another perspective, the in-depth investigation of NK cell dysfunction may also provide a rational basis for development of new strategies to harness NK cell in cancer immunotherapy.

## Clinical application of NK cell in GI cancer

With advancement of knowledge on NK cell biology, the role of NK cell in tumor immunosurveillance and its clinical implications have been extensively investigated [3]. NK cell could potentially serve as a prognostic factor for patients with GI cancer. In addition, NK cell-based immunotherapy has shown promising anti-tumor effects in a number of studies.

### NK cell-based cancer immunotherapy

In recent decades, immunotherapy has emerged as one of the most promising tools for cancer treatment. Current immunotherapy mainly focuses on cytotoxic T lymphocytes, including the CAT-T therapy and immune checkpoint inhibitors. For GI cancer, several immunotherapeutic strategies have been developed including adoptive transfer of immune cells, peptide-based vaccines, and immune checkpoint inhibitors. NK cell is endowed with a strong and specific anti-tumor potential and an immunoregulatory role of other immune cells. Harnessing NK cells could be the next frontier of GI cancer immunotherapy considering that 1) NK cells can effectively target different types of transformed cells without pre-immunization, 2) high levels of NK cells can be obtained and activated from a variety of sources, and 3) the risk of side effects such as cytokine releasing syndromes associated with T-cell-based immunotherapies could be low.

#### Adoptive NK cell transfer

**Fig. 2:**
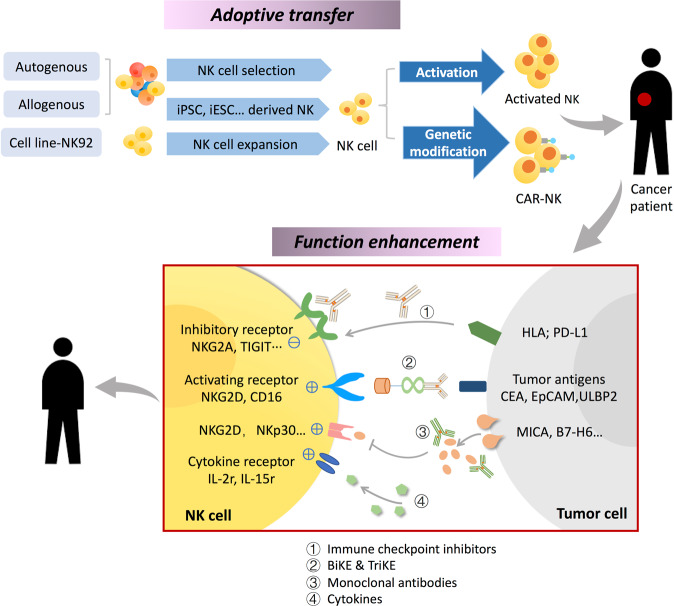
Current strategies to harness NK cell in cancer immunotherapy. The application of NK cell in cancer therapy mainly comes from two directions. **a** One is the adoptive transfer to increase the infiltration of NK cells in the tumor site. For this part, NK cells from different origins can be used, such as autologous, allogenous, the cell line and the genetic modified form (CAR-NK). Preclinical data has already shown encouraging results. **b** The other is to restore NK cell function. Immune checkpoint inhibitors, monoclonal antibodies and certain cytokines have been tried to recover or enhance the NK cell cytotoxicity. Besides, the specially designed linker that recognizes the receptors on NK cell and tumor cell at the same time can enhance the formation of NK-tumor synapse, which can increase the recognition of transformed cells by NK cells.

Since NK cells are frequently insufficient or dysfunctional in cancer patients, adoptive transfer of NK cells with anti-tumor function is an appealing aspect for cancer immunotherapy. In the initial attempt, adoptive NK cell transfer was employed to treat hematological malignancies (Fig. 2). The results demonstrated that NK cells can be harnessed and expanded in vitro and keep the cytotoxicity after the transfer. As to GI cancer, different sources of NK cells have been tried [57] (Table 1). For autologous or allogenic NK cells, expansion and activation strategies are the key points. In a phase I clinical trial (UMIN UMIN000007527), Sakamoto et al. successfully expanded NK cells to almost 4,720-fold, by stimulating peripheral blood mononuclear cell (PBMC) from GI cancer patient with OK432, IL-2 and modified FN-CH296-induced T cells. The clinical safety for transfer of expanded NK cells was further verified as no unexpected NK cell infusion-related toxicity was observed [58]. Aside from the PBMC, umbilical cord blood (UCB), human embryonic stem cells (hESCs) and induced pluripotent stem cells (iPSCs) are also precious sources to obtain NK cells with high expansion efficiency and anti-tumor function [59–61]. Xu et al. expanded NK cells from UCB by using membrane-bound interleukin-21 (IL-21). These NK cells can efficiently lyse CRC cell lines and secreted cytokines and chemokines such as interferon-γ (IFN-γ), tumor necrosis factor-α (TNF-α) and granulocyte-macrophage colony-stimulating factor (GM-CSF) [62]. FT500, the off-the-shelf NK cell derived from iPSC, can efficiently produce multiple cytokines and chemokines that help recruit and activate T cells. Combining FT500 and immune checkpoint inhibitors are presumed to combat drug resistance for cancer patients [63]. More importantly, the safety and efficiency of allogenic NK cells isolated from healthy donors have been confirmed in hematological malignancies and solid tumors [64]. Utilization of NK cell lines such as NK-92 is another choice. The advantage is that the cells are homogenous and can be easily handled and expanded. Also their cytotoxicity against tumors has been verified by in vitro and in vivo experiments [65]. Nevertheless, there is little information available about the clinical efficiency of this method. In a phase I clinical trial recruiting colon and lung cancer patients, the Hsp70-activated autologous NK cells were employed. Although enhanced NK cytotoxicity was achieved in these patients without any negative side effects, no significant clinical response was observed, which is likely due to high tumor burden and limited sample size [66]. Thus, several modified strategies have been developed for better therapeutic efficiency.

**Table 1:** Clinical trials on NK cell-based therapy related to GI cancer.

ClinicalTrials.gov Identifier	Trial	Cancer types	Sample size	Status	Phase
Adoptive transfer					
°Autologous NK cells					
°°NCT00909558	Safety and Effectiveness Study of Autologous Natural Killer and Natural Killer T Cells on Cancer	HCC, Colon cancer, Pancreatic Cancer, and other solid tumors	24	Suspended	1
°°UMIN000007527	Phase 1 clinical study of highly purified natural killer (NK) cell infusion for patients with refractory digestive/gastrointestinal cancer to standard therapy	Refractory digestive/gastrointestinal cancer	9	Completed	1
°Allogeneic NK cells					
°°NCT01147380	Safety Study of Liver Natural Killer Cell Therapy for Hepatoma Liver Transplantation	Liver Cirrhosis, HCC, Evidence of Liver Transplantation	18	Completed	1
°°NCT04162158	Safety and Efficacy of Allogeneic NK Cells Therapy in Patients With Advanced Hepatocellular Carcinoma	HCC	200	Recruiting	1/2
°°NCT03008499	High-activity Natural Killer Immunotherapy for Small Metastases of Colorectal Cancer	Metastatic CRC	20	Completed	1/2
°°NCT03008304	High-activity Natural Killer Immunotherapy for Small Metastases of Pancreatic Cancer	Pancreatic Cancer	20	Completed	1/2
°°NCT03634501	Clinical Study on Anti-tumor Effect Induced by Activated Primary Natural Killer (NK) Cells	Colon Cancer, Pancreatic Cancer, and others	200	Recruiting	1/2
°°NCT01212341	A Phase I Study of Allogeneic NK Cell Therapy in Patients With Refractory/Relapsed Lymphoma or Solid Tumor	Malignant Lymphomas Solid Tumors	18	Completed	1
°°NCT02854839	A Study of MG4101 (Allogeneic Natural Killer Cell) for Intermediate-stage of Hepatocellular Carcinoma	HCC	78	Completed	2
°°NCT02008929	To Evaluate the Efficacy and Safety of MG4101(Ex Vivo Expanded Allogeneic NK Cell)	HCC	5	Completed	2
°°NCT04106167	Long-term, Non-interventional, Observational Study Following Treatment With Fate Therapeutics FT500 Cellular Immunotherapy	GC, CRC, HCC, EGFR^+^ solid tumor, MSI, advanced solid tumor and others	76	Recruiting	
°CAR-NK					
°°NCT02839954	CAR-pNK Cell Immunotherapy in MUC1 Positive Relapsed or Refractory Solid Tumor	HCC, Pancreatic Carcinoma, CRC, GC and others	10	Unknown status	1/2
°°NCT03940820	Clinical Research of ROBO1 Specific CAR-NK Cells on Patients With Solid Tumors	Solid Tumor	20	Recruiting	1/2
°°NCT03941457	Clinical Research of ROBO1 Specific BiCAR-NK Cells on Patients With Pancreatic Cancer	Pancreatic Cancer	9	Recruiting	1/2
Reverse NK cell function					
°Checkpoint inhibitors					
°°NCT04047862	Phase 1/1b Study Investigating Safety, Tolerability, PK and Antitumor Activity of Anti-TIGIT Monoclonal Antibody BGB-A1217 in Combination With Anti-PD-1 Monoclonal Antibody Tislelizumab in Patients With Advanced Solid Tumors	Metastatic Solid Tumors	39	Recruiting	1/1b
°°NCT02671435	A Phase 1/2 Study of Durvalumab and Monalizumab in Adult Subjects With Select Advanced Solid Tumors	Advanced Solid Tumors	381	Recruiting	1/2
Combination form					
°NK cell transfer + ICIs					
°°NCT03841110	FT500 as Monotherapy and in Combination With Immune Checkpoint Inhibitors in Subjects With Advanced Solid Tumors	GC, CRC, HCC, EGFR^+^ solid tumor, MSI, advanced solid Tumor and others	76	Recruiting	1
°NK cell transfer + monoclonal antibody					
°°NCT03319459	FATE-NK100 as Monotherapy and in Combination With Monoclonal Antibody in Subjects With Advanced Solid Tumors	HER2 ^+^ GC, CRC, HCC, EGFR^+^ Solid Tumor and others	100	Active, not recruiting	1
°°NCT02805829	Combination Trastuzumab With Expanded Natural Killer Cells for Treating HER2-positive Gastric Cancer	Gastric Cancer	20	Unknown status	1/2
°°NCT02030561	NK Cell Infusions With Trastuzumab for Patients With HER2 + Breast and Gastric Cancer	Gastric cancer, breast cancer	29	Unknown status	1/2
°°NCT00720785	Natural Killer Cells and Bortezomib to Treat Cancer	Pancreatic cancer, CRC, Chronic myeloid leukemia and others	61	Recruiting	1
°NK cell-based immunotherapy + Locoregional treatment					
°°NCT03008343	Combination of Irreversible Electroporation and NK Immunotherapy for Recurrent Liver Cancer	Recurrent liver carcinoma	20	Completed	1/2
°°NCT02718859	Combination of Irreversible Electroporation and NK Immunotherapy for Advanced Pancreatic Cancer	Pancreatic Cancer	60	Completed	1/2
°°NCT02849015	Combination of Cryosurgery and NK Immunotherapy for Tumors in Transplanted Liver	Liver Tumor/Evidence of Liver Transplantation	20	Completed	1/2
°°NCT02843802	Combination of Cryosurgery and NK Immunotherapy for Advanced Liver Cancer	Secondary Malignant Neoplasm of Liver	30	Completed	1/2
°°NCT02843581	Combination of Cryosurgery and NK Immunotherapy for Advanced Esophageal Cancer	Metastatic Esophageal Cancer	60	Completed	1/2
°Cytokine + chemotherapy					
°°NCT02559674	Phase Ib/II Study of ALT-803 in Combination With Gemcitabine and Nab-paclitaxel in Patients With Advanced Pancreatic Cancer	Advanced Pancreatic Cancer	8	Completed	1

Inspired by the chimeric antigen receptor- T-cell (CAR-T) therapy, it is feasible to develop CAR-modified NK cells for immunotherapy (Fig. 2). Li et al. infused the CARs on the iPSC-derived NK cells to generate NK-CAR iPSC-NK cells which exerted enhanced expansion efficiency and cytotoxicity compared with the iPSC-NK and PB-NK (PBMC-derived NK cell) [67]. In a clinical trial with CRC patients, NKG2D-CAR-NK cells were constructed by transducing the NK cells with NKG2D-CAR. Varying degrees of clinical remission were observed in patients treated with NKG2D-CAR-NK, indicating that CAR-NK is promising for cancer therapy [68]. Moreover, the NK-92 cell line modified with bispecific chimeric PD1-DAP10/NKG2D shown enhanced cytotoxicity in vitro and tumor control in vivo [69]. Another human NK cell line YT is modified with chimeric immunoglobulin T-cell receptor specially recognizing human carcinoembryonic antigen (CEA), which is frequently overexpressed in GI cancers such as colorectal, gastric, and pancreatic carcinomas. Both in vitro and in vivo experiment confirmed the improved cytotoxicity of the modified NK cells [70]. To date, there are three Phase 1/2 clinical trials ongoing using CAR-NK to treat GI cancer (Table 1: NCT02839954; NCT03941457; NCT03940820). Compared with the CAR-T therapy, recent investigation found that CAR-NK does not cause serious GVHD (graft-versus-host disease) and CRS (cytokine release syndrome) [71], as NK cells have initial cytotoxicity to transformed cells without pre-immunization. This provides solid grounds for further research into the CAR-NK in cancer therapy.

#### Reverse the NK cell dysfunction

Adequate immune cell infiltration and normal function are the two prerequisites for the successful immune surveillance. The adoptive transfer only mobilizes the immune cells in the fight against tumor. However, cancer cells have developed multiple mechanisms to escape from the immune surveillance and attack. The function of NK cells is frequently impaired in cancers. Therefore, reversing the NK cell dysfunction is another strategy in NK cell-based cancer immunotherapy. Until now, multiple drugs have been developed to reverse the impaired NK cell function, including the immune checkpoint inhibitors, monoclonal antibodies, cytokines and the specifically designed linkers (Fig. 2).

##### Immune checkpoint inhibitors

The immune checkpoints are molecules acting as a brake on immune cells and balancing host immune system. Unfortunately, cancer cells can take advantage of checkpoints such as CTLA-4, PD-1, TIGIT and indoleamine 2, 3-dioxygenase 1 (IDO1) to evade the immune response. The advent of immune checkpoint inhibitors (ICIs), which substantially enhances host anti-tumor immunity, represents a major hallmark in cancer immunotherapy. Multiple investigations have uncovered the inhibitory checkpoint inhibitors expressed on NK cells in tumor microenvironment, including the famous PD-1, CTLA-4 as well as newly identified NKG2A, TIGIT [44, 72], which are now appreciated as promising targets for NK cells-based immunotherapy. Moreover, shifting the balance between activating and inhibitory receptors of NK cells can further enhance the activity of NK cells, prompting scientists to embark on multiple clinical efforts to assess the safety and feasibility of NK cell-based ICIs. The initial attempt was carried out in hematological malignancies. The results demonstrated that blocking of inhibitory receptors can enhance NK cells’ cytotoxicity towards the tumor cells. In animal models, NK cells were found to make contributions to the disease remission in response to anti-PD-1/PD-L1 treatment [73]. Also, the antibody Monalizumab that was developed to target inhibitory receptor NKG2A exhibited encouraging anti-tumor effects [74].

##### Monoclonal antibody

Monoclonal antibody targeting specific pathways or protein is one direction for cancer therapy. Many FDA-approved monoclonal antibodies, e.g., Cetuximab, have been found to partially depend on NK cells [75], indicating a new direction to reverse NK cell dysfunction and block tumor-mediated immunosuppression. GA201, the modified glyco-engineered anti-EGFR mAb, has shown enhanced tumor-suppressive effect through NK cell-mediated ADCC in CRC patients. The therapeutic efficiency was observed in several patients, especially those with poor response to traditional Cetuximab [76]. The CEACAM5, which is frequently overexpressed in gastrointestinal cancer such as colorectal, gastric, and pancreatic cancer, is closely related to impaired NK cell function through interaction with CEACAM1 [77]. Preclinical and Phase I clinical trials have been conducted to evaluate the safety and efficacy of anti-CEACAM5 antibody in CRC [78].

##### Cytokines

A large number of cytokines are involved in shaping tumor microenvironment and regulating immune response against tumor. In preclinical studies, the MHC-I deficient tumors responded better to treatment of cytokine such as IL-12 and IL-18, raising the question of whether the efficiency of cytokine therapy relies on NK cells, considering the well-known role of NK cells in targeting MHC-I deficient tumor [79]. A series of studies demonstrated that the cytokine treatment can markedly enhance the function of NK cells both in vitro and in vivo. For example, ex vivo treatment with IL-2 can restore the Herceptin-mediated ADCC function of NK cells derived from gastric cancer patients [80]. In addition, IL-15 can partly promote the maturation and function of NK cells. IL-15 administration in CRC patients was found to activate the function of infiltrating NK cells in liver metastases [81]. In keeping with this, enhanced NK cell proliferation and cytokine production was observed in patients [82]. In multiple clinical trials, ALT-803, an IL-15 superagonist complex, exhibited a more specific and strong effect in promoting the proliferation and cytotoxicity of NK cells [83]. The cytokine-based treatment is often combined with other types of therapy. For example, ALT-803 plus traditional chemotherapy is now tested in a clinical trial recruiting a small number of patients with advanced pancreatic cancer [84].

##### BiKE and TriKE

From the antibody-dependent cell-mediated cytotoxicity (ADCC) of NK cell function mechanism, specially designed bi- and tri-specific killer engagers (BiKEs and TriKEs), the small molecules linking a single-chain Fv against CD16 that is expressed on the NK cell with one (BiKE) or two (TriKE) tumor-associated antigens, were developed to improve the formation of immunological synapses between NK cells and tumor cells. For example, tumor-associated antigen CD133 and EpCAM have been used to generate BiKEs. The CD133/CD16 BiKE can boost the cytotoxicity of NK cells against CD133^+^ CRC stem cells [85]. Similarly, EpCAM/CD16 BiKE increases the synapse formation between NK cells and human cancer cells including colon, neck and breast cancers [86]. In line with these findings, ULBP2-aCEA BiKE promotes the recognition and lysis of CEA^+^ tumor cells by the NK cells through NKG2D-ULBP2 interaction, and the efficiency against colon cancer was verified using animal models [87]. Moreover, optimized TriKE shown stronger NK cell mediated cytotoxicity by targeting two activating receptors on NK cells, NKp46 and CD16, and a tumor antigen on cancer cells [88, 89]. This multi-targets antibody exhibits sufficient activity and safety in animal experiments. Although most of the bispecific engagers are now at the preclinical stage, their ability to enhance NK cell-mediated cytotoxicity against targets hold substantial promise for treating cancer patients. Further investigations evaluating the safety and efficiency of BiKE and TriKE are warranted before clinical use.

#### Combination therapy to enhance the NK cell cytotoxicity

There is increasing consensus that using combination therapy can achieve better clinical response with less side effects. Inspired by the combination of anti-PD-1 and anti-CTLA4 in lung cancer, the combination of different checkpoint inhibitors (anti-PD-1, anti-NKG2A and anti-TIGIT) was investigated and synergistic anti-tumor effect was observed in preclinical experiments [72]. For example, the metastatic microsatellite stable (MSS) CRC patients are not good responders to the anti-PD-1 immunotherapy such as Pembrolizumab, but in the phase 1 clinical trial (NCT02671435) combining the Monalizumab (anti-NKG2A antibody) and Durvalumab (anti-PD-L1 antibody), three out of 39 patients were evaluated as partial response (PR) and 19 patients as stable disease (SD). No fatal adverse event (AEs) or AEs induced drop out were reported. Combining the NK cell-based immune-therapy with current ICIS maybe one direction to improve the clinical response.

Different combination strategies have been tried to achieve a much stronger clinical response. For example, combing the adoptive NK cell transfer and monoclonal antibody therapy is one common combination form. Cetuximab is commonly used in CRC patients, enhanced anti-tumor activity and improved clinical outcome was observed in CRC patients when combined with NK cell adoptive transfer [90]. In phase 1 clinical trial, four out of nine patients with advanced colon cancer achieved clinical benefit. Selected immune parameters were monitored during the therapy and anti-tumor immune responses were improved as exemplified by increased IFN‐γ production and reduced number of peripheral regulatory T cells (Tregs) [91]. Similarly, markedly increased anti-tumor effects were observed when the monoclonal antibody Regorafenib was combined with CAR-NK-92 cells in colon cancer mouse models [92]. An additional study was carried out to evaluate the efficacy of traditional chemotherapy combined with NK cells-based immunotherapy in patients with locally advanced colon carcinoma. Both the 5-year progression-free survival (PFS) and overall survival (OS) rates increased significantly (51.1% versus 35%, *P* = 0.044; 72.5% versus 51.6%, *P* = 0.037, respectively) in combination group without any unacceptable side effects [93]. To date, many clinical trials for combination therapy in GI cancer are ongoing (Table 1).

Preclinical experiments have achieved encouraging results, highlighting the potential of combination therapy in cancer immunotherapy. However, the in vitro and animal experiments often fail to mimic the complex nature of human immunity, which has been regarded as a huge challenge for immunotherapy. Further clinical investigations are warranted to determine how to better harness NK cell in combination with other cancer therapies.

### Prognostic significance of NK cell in GI cancer

A large body of evidence suggests that the infiltration of functional NK cells is closely correlated with cancer risk, cancer stage, and patient prognosis among different cancer types. The first evidence came from the 11-year follow up study conducted on the general population (*n* = 3625) in Japan showing that higher natural cytotoxicity of peripheral lymphocytes, the majority of which comes from NK cells, was associated with lower cancer risk [19]. Subsequently, numerous clinical trials have been carried out to explore the relationship between NK cell activity and cancer stage and clinical outcome in GI cancers (Table 2). Consistently, low infiltration of functional NK cells predicted advanced disease stage, more metastasis and post-operative recurrence, and poor survival in multiple cohorts of GI cancer patients including GC, HCC, CRC, and esophageal cancer [94, 95]. More importantly, the number and function of peripheral NK cells also denote diagnostic and prognostic value for CRC patients, offering a feasible way for detection of NK cells in clinical application [96].

**Table 2:** Clinical trials on the investigation for the diagnosis value of NK cell.

ClinicalTrials.gov Identifier	Trial	Cancer types	Parameter analysis	Sample size	status
NCT03422120	Human Blood Specimen Collection to Evaluate Immune Cell Function	CRC	NK cell Activity (baseline vs. after surgery)	69	Completed
NCT03289988	Novel Blood-based Colorectal Cancer Screening Method Using Natural Killer Cell Activity and Gene Panel Expression	Colorectal Adenoma and CRC	NK cell activity and gene panel expression analysis (blood sample)	964	Recruiting
NCT02869269	A Study of Circulating Immune Cell Activity Changes in Blood of Colorectal Cancer Patients	CRC	Circulating helper T cell, cytotoxic T cells and NK cell activity	67	Completed
NCT03665571	Evaluation of Killing Activity of Expanded Natural Killer Cells From Blood in Patients With Pancreatic Cancer	Pancreatic Neoplasms	NK cell activity(receptor specific activation method)	100	Recruiting
NCT02615665	Intratumoral CD3^+^ and NKp46^+^ Cells in Colorectal Liver Metastases	CRC with liver metastases	CD3^+^ and NKp46^+^ Cells analysis (liver resection)	121	Completed
NCT02557061	Prognostic Value of the Lymphocytic Infiltrate in Colon Cancers	CRC	Blood sample (surgery) analysis	56	Completed
NCT02291198	Measurement of NK Cell Activity in Whole Blood in Subjects Being Screened for Colorectal Cancer Using Colonoscopy	Subjects screened for CRC via colonoscopy	NK cell activity (blood sample)	1081	Completed
NCT02887599	Assessment of the Cytotoxic Immune Status of Pancreatic Cancer Patients and the Severity of the Cancer Using Measurement of Natural Killer Cell Activities	Pancreatic Cancer	Quantitative measurements of NK cell activities	203	Completed

NK cell activity testing is useful in monitoring anti-tumor immunity and in predicting patient outcome. However, there are some difficulties with detection of such parameter in clinical practice, e.g., tissue biopsy and adequate pre-culture of target cells with NK cells are required. In addition, the accuracy of this method remains suboptimal. Finding a surrogate marker that can be easily detected and robustly reflect the activity of NK cells in vivo is required for further clinical application. Recently, MICA, the ligand of human NK cell activating receptor NKG2D, is identified as a promising biomarker for cancer. In HCC, the soluble form of MICA (sMICA) in serum is negatively associated with NKG2D expression level on NK cells [41]. Consistent results were obtained in the colon and pancreatic cancer [97], implying that serum MICA could be used as a prognostic biomarker for GI cancer patients. Likewise, in patients with advanced HCC, NKp30, another activating receptor of NK cells, is found to be downregulated by serum B7-H6 released from tumor cells [98]. Moreover, intratumoral IL-37 expression was positively correlated with infiltrating CD57^+^ NK cells, and higher IL-37 expression predicted smaller tumor size and better survival for HCC patients [99]. Further investigation into the above mentioned molecules and cytokines is necessary before implementation of these findings into clinical practice.

## Conclusion and perspective

With the great success of immune checkpoint inhibitors represented by anti-PD1/PD-L1 and anti-CTLA4 in multiple cancers, the T-cell-centered immunotherapy posed a milestone for cancer treatment. More importantly, it provides us with a novel direction of inducing robust immune responses to tumors, which is superior to traditional chemotherapy as there is better therapeutic efficiency and is associated with less toxicities and debilitating side effects. Originally starting from the cytotoxic T lymphocytes, many other immune cells later attracted scientists’ interests in the field of cancer immunity. The exponentially growing understanding of NK cell unveiled the fact that this natural cytotoxic innate immune cell plays a vital role in cancer immune surveillance via modulation of both the innate and adaptive immunity. Also, the similarities between NK cells and T cells further indicate the great promise that NK cells hold in cancer therapy. Harnessing NK cell in cancer immunotherapy is an intriguing idea, leading to the development of numerous clinical trials, albeit multiple difficulties being encountered.

Late diagnosis, poor therapeutic efficiency, and high recurrence rate make GI cancer one of the major causes of cancer-related mortalities worldwide. There is continued need to improve therapeutic efficiency and early diagnosis. However, the current attempts on the FDA-approved immune agents such as Pembrolizumab in GI malignancies did not show very encouraging results as in melanoma and lung cancer. Further investigations are needed for better manipulation of the immune system to improve the therapeutic efficiency. Here we summarized the current investigations on NK cell in GI malignancies. Compelling evidence has shown that NK cells are involved in GI cancer, ranging from the human data to animal model-based mechanism investigation. However, we are just at the beginning of immunotherapy in GI cancer. For the NK-cell-based immunotherapy, the particular challenge is to successfully translate the current knowledge into clinical GI cancer treatment. Positive results are expected for the ongoing clinical trials. Finally, considering the close interplay of the innate and adaptive immunity and complex interactions among different kinds of immune cells, future studies will need to address the gaps on how to combine different types of immune cells in the treatment strategies to achieve synthetic anti-tumor immunity, and hence better clinical response.
